# Metabolic and senescence characteristics associated with the immune microenvironment in non-small cell lung cancer: insights from single-cell RNA sequencing

**DOI:** 10.18632/aging.205146

**Published:** 2023-10-26

**Authors:** Hongliang Liao, Zihao Wan, Yaqin Liang, Lin Kang, Renping Wan

**Affiliations:** 1 Department of Thoracic Surgery, The Yuebei People’s Hospital of Shaoguan, Shaoguan, Guangdong 512025, China; 2 College of Physical Education and Health, Guangzhou University of Chinese Medicine, Guangzhou, Guangdong, China; 3 Department of Nursing Medical College, Shaoguan University, Shaoguan, Guangdong 512005, China; 4 Department of Gynaecology and Obstetrics, The Qujiang District Maternal and Child Health Care Hospital, Shaoguan, Guangdong, China

**Keywords:** immune cell, single cell, TME, T cell, NSCLC, cell phenotype

## Abstract

Non-small lung cancer (NSCLC) has been defined as a highly life-threatening heterogeneous disease, with high mortality and occurrence. Recent research has indicated that tumor-infiltrating lymphocytes play a key determinant role in cancer progression. Emerging single-cell RNA sequencing (also termed scRNA-seq) has been extensively applied to depict the baseline landscape of the cell composition and function phenotype in the tumor environment (TME). Herein, we dissected the cell types in NSCLC samples (including tissue and blood) and identified three types of cell marker genes including cancer cells, T cells, and macrophages by integrating two NSCLC-associated scRNA-seq datasets in GEO. Survival analysis indicated that 17 marker genes were related to tumor prognosis. Function annotation was used to scrutinize the molecular mechanism of these marker genes in different cells. Besides, we investigated the developmental trajectory and T cell receptor repertoire diversity of tumor-infiltrating T cells. Our analysis will help further understand the complexity of cell components and the heterogeneity of TME in NSCLC.

## INTRODUCTION

Non-small cell lung cancer (NSCLC) is the leading life-threatening cancer worldwide and accounts for approximately 85% of lung cancers [1, 2]. NSCLC tumors generally harbor extensive genomic variations, with broad alternation load associating with better response to checkpoint blockade therapies, albeit with some exceptions [3, 4]. With the rapid development of surgical and chemotherapeutical therapies, overall survival has improved considerably; however, clinical outcomes in advanced NSCLC patients remain unfavorable [5]. The past several decades have witnessed a revolution in cancer treatment by moving away from target tumors typically (e.g., radiotherapy, and chemotherapy), although with great advancement in improving the prognosis of NSCLC, toward antibody-based immunotherapy that nudges immune responses against tumors [6, 7].

Recent advances in single-cell sequencing technology have provided a powerful tool for basic research and hold significant potential for deciphering biological systems with unprecedented resolution [8]. Compared with traditional bulk sequencing, the most unique feature of single-cell sequencing lies in the analysis of intercellular heterogeneity and the requirement of obtaining single cells in good status [9, 10]. Sequencing the genome of individual cells, encompassing transcriptomic and epigenomic assays, is promising to reveal somatic mutations and comprehensively characterize the diversity of tumor cell types [11]. The lung tumor microenvironment, comprised of tumor bulk plus supporting cells, plays a critical role in the onset, progression, and malignant transformation of NSCLC and is tightly correlated to therapeutic outcomes [12]. Single-cell DNA sequencing can delineate the genomic variations within a single cell, but it fails to identify salient expression differences in heterogeneous cells [13, 14]. However, the generation of transcriptional profiles of single cells by single-cell RNA sequencing (scRNA-seq) enables the investigation of intercellular heterogeneity on a transcriptome-wide and single nucleotide and level [15]. scRNA-seq of NSCLC samples can disclose the intrinsic gene regulatory mechanisms that determine cellular properties and reveal the developmental and evolutionary relationships of NSCLC cell populations. In addition, it contributes to dissect the correlation between NSCLC heterogeneity, signaling pathways, drug resistance and microenvironment shaping, which is of great importance for NSCLC treatment.

In the current study, we investigated the cell types and cell features genes in TME of NSCLC by integrating the two scRNA datasets of NSCLC from GEO. Also, we studied the association between cell marker genes and tumor survival. Of note, we analyzed the single-cell trajectory features and characteristic differences of T cell receptors (TCR) in NSCLC. Our research highlighted the decisive function of the cell component of TME and emphasized the presence of TME heterogeneity in tumor progression.

## MATERIALS AND METHODS

### Data collection and processing

The original scRNA-seq gene expression matrix information related to NSCLC was downloaded from the GEO database. We selected GSE162498 with 17 samples (13 tumor samples and 4 blood samples) and GSE117570 with 8 samples (4 tumor samples and 4 paracancerous samples) as our metadata. The single cell TCR-seq (T cell receptor) annotation file was acquired from GSE162498 with 10 samples (6 tumor samples, 2 para-tumor normal samples, and 4 blood samples). The workflow of data processing was performed according to previous research [16]. Generally, the cells that meet the requirements are screened out according to the following standard: min. features = 100, min. cells = 10, nFeature RNA > 100 and nFeature RNA < 5000, percent.mito < 20, nCount RNA > 10. There was a batch effect between GSE117570 and GSE162498. Therefore, we removed the batch effect by using FindAnchor and IntegrateData functions in the Seurat package (V4) in R software [17]. By integrating GSE117570 and GSE162498 data, a total of 23886 genes in 153,595 cells were identified.

### Cell cluster analysis

Principal component analysis (PCA) is a linear method used to reduce data complex dimensionality. PCA analysis is to condense the information of a large number of genes in the data into a few variables (representing the main effects in the sample). Then, just 2–3 variables (named PC1, PC2, and PC3) can represent most of the information contained in tens of thousands of genes. Then the difference in expression between cells is reflected in the differences in the values of PC1 and PC2 [18]. t-distributed Stochastic Neighbor Embedding (t-SNE) algorithm is a machine learning algorithm for dimensionality reduction, which is used for exploring high-dimensional data [19]. Uniform Manifold Approximation and Projection (UMAP), is an emerging dimensionality reduction algorithm, which can retain the features of the original data to the maximum extent and reduce the feature dimension [20]. We normalize the scRNA-seq data by using the Seurat package. After the dimension of PCA, a total of 15w cells were clustered into 29 clusters by tSNE/UMAP analysis. Among that, the number of cells in the tumor sample was the highest (113448 cells) and normal was the lowest (6432 cells).

### Identification of cell marker genes

The specific gene list expressed in each cluster was collected by using the FindAllMarkers function in the Seurat package. Herein, the cell type in each cluster was annotated by the combination of the singleR (3.17) method and manual annotation (Supplementary Table 1). The marker gene annotated manually comes from this article [21] and the immune cell marker website (http://www.cellsignal.com/pathways/immune-cell-markers-human). Also, these cluster-specific genes identified by FindAllMarkers were regarded as cell type-specific genes of annotated cells in the matching cluster. In each cell type, the differentially expressed genes (DEGs) among different groups (tumor, normal, and blood samples) were acquired by using the limma and DESeq method with the threshold of *p*-value < 0.05. The overlapping genes between cell-specific genes and DEGs were defined as the matching cell marker genes.

### Survival analysis and gene expression pattern of marker genes

The influence of cell marker genes on NSCLC prognosis was assessed based on a Cox model, which was created to estimate the survival risk of patients under different conditions. Kaplan-Meier curve analysis was performed to compare the survival differences in patients with different marker gene expression contexts by using the survival (3.5.5) package in R.

### Function enrichment and ssGSEA analysis

GO and KEGG enrichment analysis of cell marker genes was used to identify the major biological function and involved signaling pathways of each type of cell. In addition, we also conducted the gene set enrichment analysis to further explore the predominant signaling axis implanted by each type of cell according to previous research. ssGSEA was termed as a non-parametric and unsupervised approach, which was used to calculate the normalized enrichment score of T cell subsets in our metadata with reference to the marker genes of 17 T cell subsets.

### Hallmark of cancer and immune signature analysis

We downloaded the hallmark gene set and immunologic signature gene set from the MSigDB database (http://www.gsea-msigdb.org/gsea/msigdb/). The GSEA method was used to analyze the predominant molecular signals of cell marker genes in tumor-associated pathways and immune-related pathways.

### Trajectory analysis

We arranged T cells according to pseudotime along a trajectory with the orderCells function by using the monocle3 method. Then we conducted a data dimensionality analysis of cell clustering by using the DDRTree approach. After that, the trajectory image of T cells was visualized by UMAP. Here we defined the naïve T cell as the root node. Likewise, the trajectories of myeloid cells are analyzed with monocle3, and the node at the bifurcation is selected as the root node.

### TCR analysis

The T-cell receptor sequencing (TCR-seq) technique was applied to comprehensively and rapidly detect the TCR diversity of antigen recognition decisive surface molecules. When the immune response occurs, the gene rearrangement in the VDJ region of TCR formed many different immune receptors, that is, the immune library. The main purpose of TCR-seq data analysis was to count the occurrence frequency of genes in different regions, namely geneUsage. Here we analyzed the distribution pattern of TCR by using the immunarch package in R based on GSE16249. The results were visualized by the vis function in ggplot2 (3.4.3).

### Availability of data and materials

The datasets used and/or analyzed in the present study are available from the corresponding author on reasonable request. R code script was shown in Supplementary File 1.

## RESULTS

### The cell distribution pattern in NSCLC

**Figure 1:**
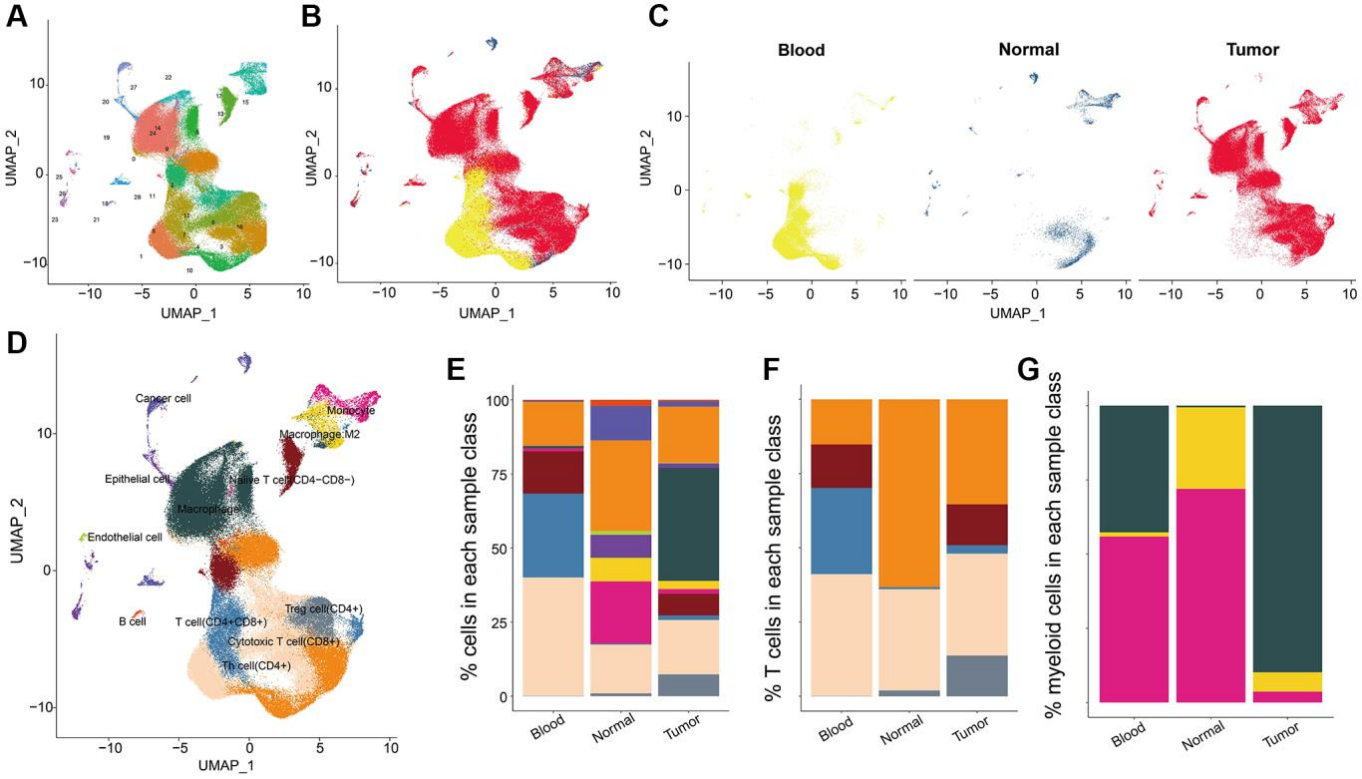
**The cell distribution pattern in NSCLC.** (**A**) The cell cluster analysis of scRNA data. (**B**, **C**) The cell distribution in three sample types (tumor tissues, para-tumor normal tissues, and blood samples). (**D**) Cell annotation results of UMAP cluster analysis. (**E**) Percentages of clustered total cells in three types of samples including blood, normal tissue and tumor tissue group. (**F**) The cell percentages of clustered T cells in three types of samples. (**G**) The cell percentages of clustered macrophage subpopulations in three types of samples.

We identified a total of 23886 genes in 153,595 cells by integrating the GSE117570 and GSE162498 data with Seurat algorism. According to tSNE/UMAP analysis, these cells were mainly enriched into 29 distinct clusters (Figure 1A). Also, the cell distribution pattern in three sample types (tumor tissues, corresponding para-tumor normal tissues, and blood samples) was visualized. Most cells were mainly augmented in tumor tissue samples while the corresponding para-tumor normal tissue samples gained the lowest cell abundance (Figure 1B, 1C). The overall cell could be annotated into 12 cell types (Figure 1D). The cellular compositions, including cancer cells, T cells, B cells, endothelial cells, and so on (a total of 12 types of cells) in each sample type differed from other types. We found that blood samples had the highest abundance of T cells with the highest percentage of CD4+ T cells. Tumor tissue carried most cellular components such as cancer cells, T cells, B cells, and endothelial cells, among which macrophage had the highest percentages, following CD4+ and CD8+ T cells (Figure 1E). Furthermore, we found that CD4+ and CD8+ T cells were evenly distributed in tumor samples compared with the normal group (Figure 1F). Consistently, the macrophage was the most abundant of three myeloid cells (macrophage, monocyte, and M2-macrophage) in tumor tissues compared to the normal tissue (Figure 1G). These results suggested the active anti-cancer response in tumor tissues.

### Gene expression features in cell clusters

**Figure 2:**
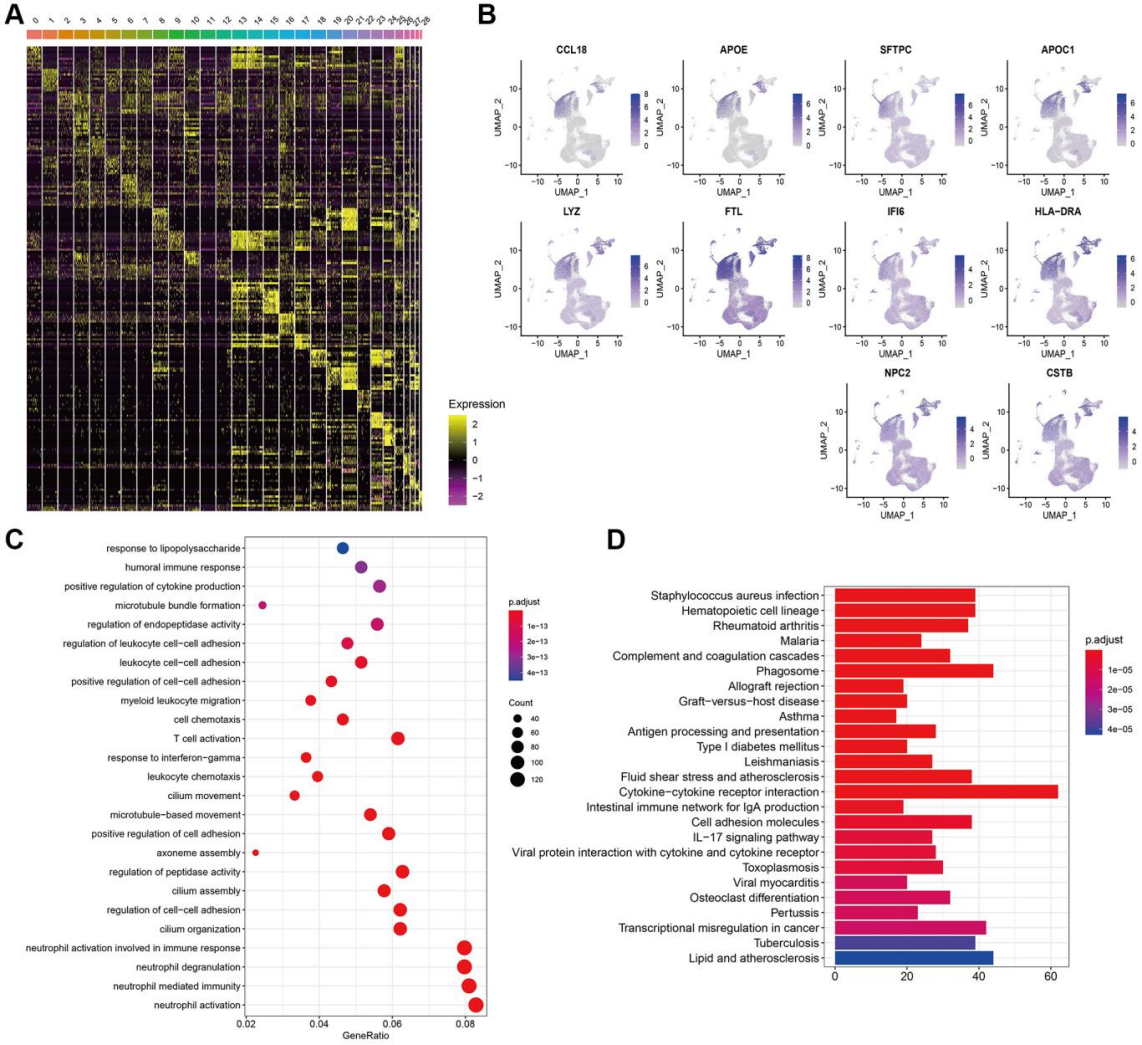
**Gene expression features in cell clusters.** (**A**) Heatmap of the top 10 DEGs in 29 clusters. (**B**) Expression of top 10 DEGs of cluster 0 in 29 clusters. (**C**, **D**) GO and KEGG analysis of DEGs in cluster 0.

The heat map of the top 10 DEGs in each cluster was shown in Figure 2A. Interestingly, the top 10 DEGs identified by cluster 0 were distributed at cluster 0 other than resident clusters (Figure 2B), validating the efficacy of our cell cluster analysis. Observing the abundance composition of cluster 0, we obtained DEGs in cluster 0. GO and KEGG analysis of DEGs in cluster 0 was displayed in Figure 2C, 2D. They were associated with T cell activation and autoimmune disease pathways.

### Identification of prognostic associated marker genes (PAMGs) of cells

**Figure 3:**
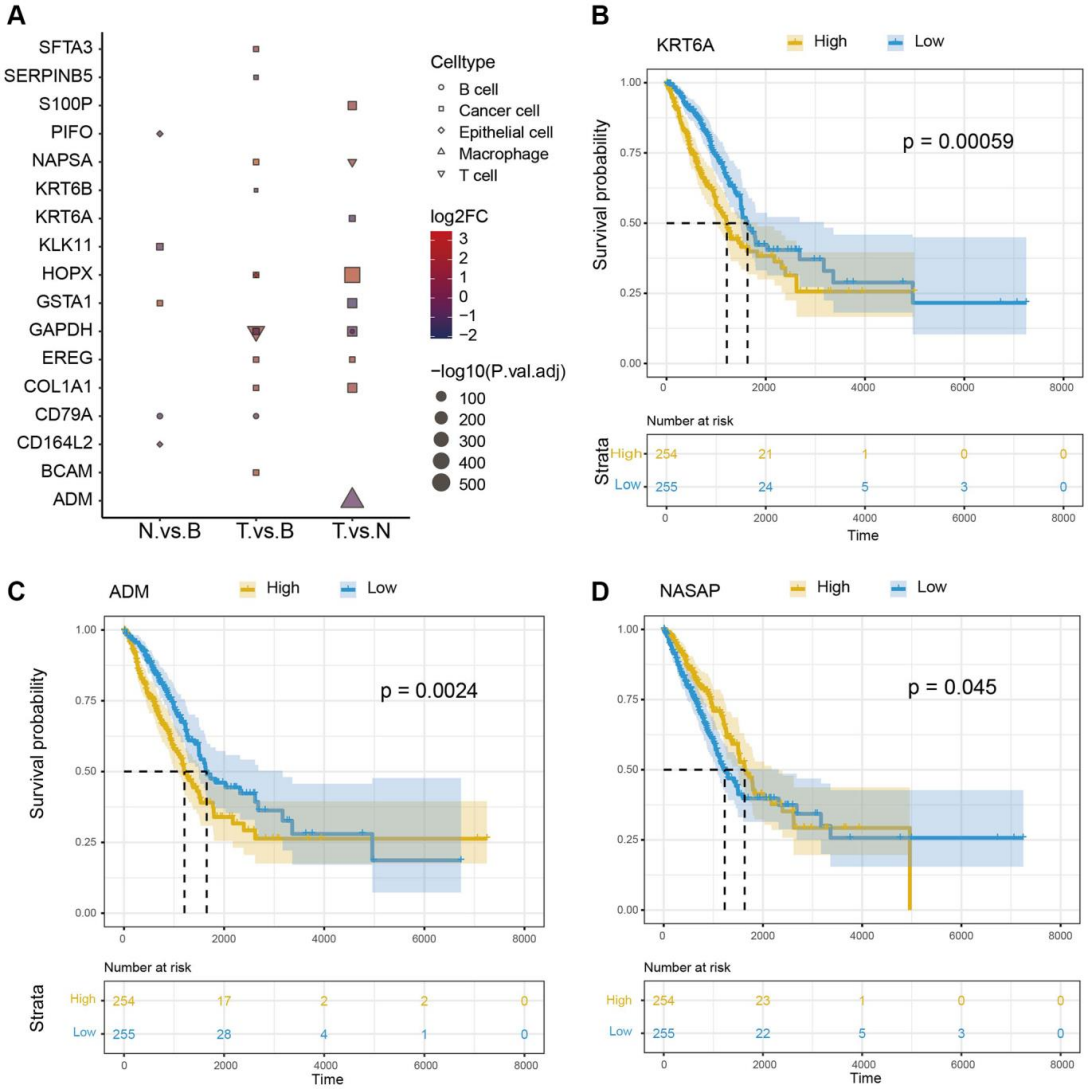
**Survival analysis of cell marker genes.** (**A**) DEGs between different groups. (**B**–**D**) Survival analysis of 3 representative marker genes (KRT6A, NASPA, and ADM).

We obtained the DEGs between three samples of each cell type. The top 10 DEGs in were shown in Figure 3A. The overlapping genes between DEGs and cell-specific gene lists were used as marker genes for matching cells. 285 marker genes were identified in the cancer cell, which was consistent with the tumor tissue feature. 142 marker genes were identified in T cells and 225 marker genes in macrophages. Here, we acquired cancer cells, T cells, and macrophage marker genes. Furthermore, we investigated the influence of these cell marker genes on NSCLC survival based on TCGA-LC clinical data. Results indicated that 17 marker genes were found to have significant effects on tumor survival (3 representative genes, KRT6A, NASPA, and ADM Figure 3B–3D).

### Expression pattern of PAMGs in different types of cells

**Figure 4:**
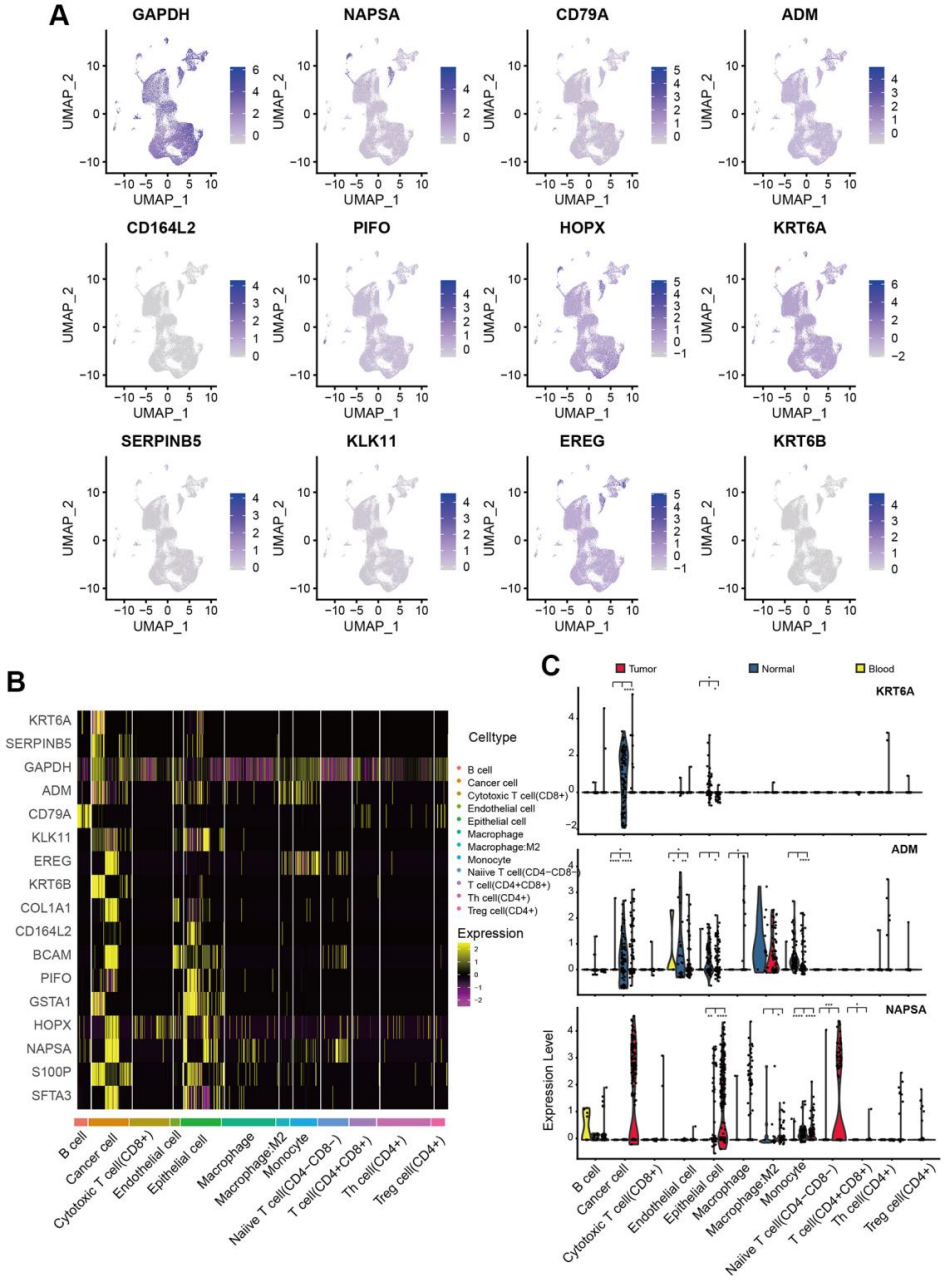
**Expression pattern of PAMGs in different types of cells.** (**A**) Expression of 17 PAMGs in three sample types. (**B**) Heatmap of 17 PAMGs in different types of cells. (**C**) Box plot of 3 representative genes expression (KRT6A, NASPA, and ADM) among tumor, blood, and normal tissues.

Considering these PAMGs in tumor prognosis and development, we also explored their expression pattern in our training set (GSE117570 and GSE162498). Gene expression patterns of 17 PAMGs in three sample types were shown in Figure 4A. The heatmap of 17 PAMGs in different types of cells was displayed in Figure 4B. Furthermore, the box plots of gene expression of 3 representative genes, KRT6A, NASPA, and ADM were also displayed in Figure 4C. KRT6A was mainly expressed in the cancer cell. We could conclude that most PAMGs were up-regulated in cancer cells and epithelial cells. These results demonstrated that cancer cells play a crucial role in cancer progression while other cells such as T cells were not directly engaged in tumor development.

### Enrichment analysis of marker genes

**Figure 5:**
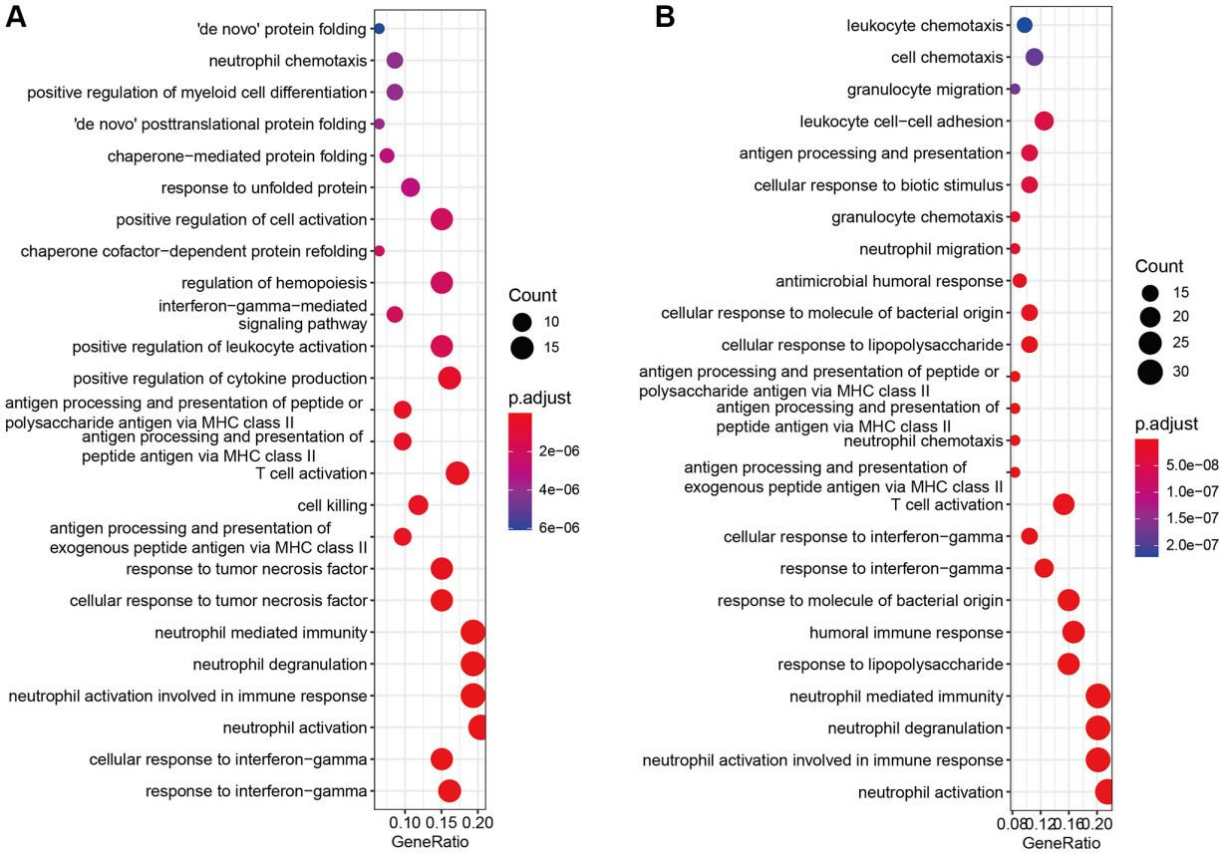
**Enrichment analysis of marker genes.** (**A**, **B**) GO and KEGG enrichment analysis of cell marker genes.

The GO and KEGG enrichment analysis of cell marker genes in different cell types were performed to examine the functional difference. The most significant pathway in T cells was “response to interferon-gamma”, which was found to be related to antigen recognition (Figure 5A). Recent studies have revealed that lung cancer–specific tumor-infiltrating CD8+ T cells had a distinctive differentiation trajectory, which acquired effector and exhausted phenotypes. This unconventional T cell dysfunction explained the ICB resistance for some NSCLC patients [22]. Of course, IFN-γ has a pivotal influence on ICB efficacy, evidenced by that IFN-γ-related mRNA profile exerted a predictive influence on PD-1 blockade response in a clinical trial setting [23]. On the contrary, the most significant pathway enriched in macrophage was neutrophil activation (Figure 5B), which is related to innate immune activation. Increasing data has demonstrated that there were two lineages of macrophages: local tissue-resident macrophages and those derived from monocytes in NSCLC [24]. Early-stage tumors were influenced by tissue-resident macrophages which enhance tumor cell invasiveness and create a protective shield by triggering a powerful regulatory T cell response [24]. Interestingly, as the tumor expands, monocyte-derived macrophages rise in number, pushing tissue-resident macrophages to periphery of the TME [24]. These data highlighted the potential in focusing on tissue-resident macrophages for early NSCLC interventions. These results indicated that the gene expression pattern of maker genes was strictly governed by the cell functional phenotypes.

### Hallmark and immune signature analysis of marker genes

**Figure 6:**
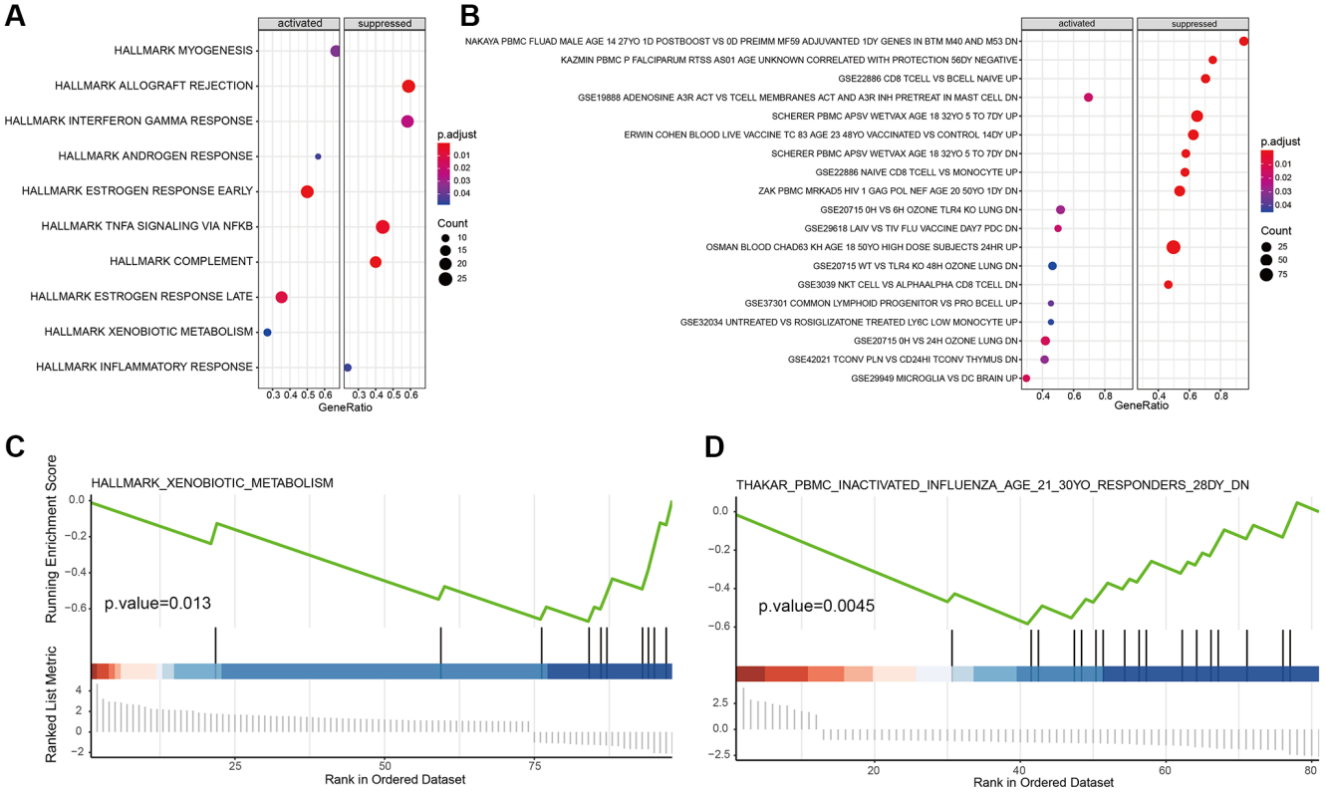
**Hallmark and immune signature analysis of marker genes.** (**A**, **B**) KEGG analysis of B cell marker genes in tumor-associated signal pathways and immune signature pathway. (**C**, **D**) GSEA results of cancer cell marker and macrophage marker genes.

We downloaded the hallmark and immunologic signature and gene set from the MSigDB database to scrutinize the function of our marker genes in tumor-associated and immune-related pathways. By KEGG analysis in tumor signal pathways, we found that B cell maker genes were mainly enriched in estrogen response, interferon-gamma response, androgen response, TNF-α signaling via NK-κB, and complement response (Figure 6A). The immune signature pathway of B cell maker genes was shown in Figure 6B. GSEA results illustrated that the cancer cell marker gene was significantly enriched in xenobiotic metabolism (Figure 6C), suggesting the hyperactive metabolism response of cancer cells. Also, GSEA of immune signature enrichment showed that the macrophage marker gene was mainly engaged in influenza induced by PBMC inactivation (Figure 6D), suggesting macrophages play a key role in innate immunity. This was consistent with our previous results (Figure 4B).

### Identification of T cell subpopulations

**Figure 7:**
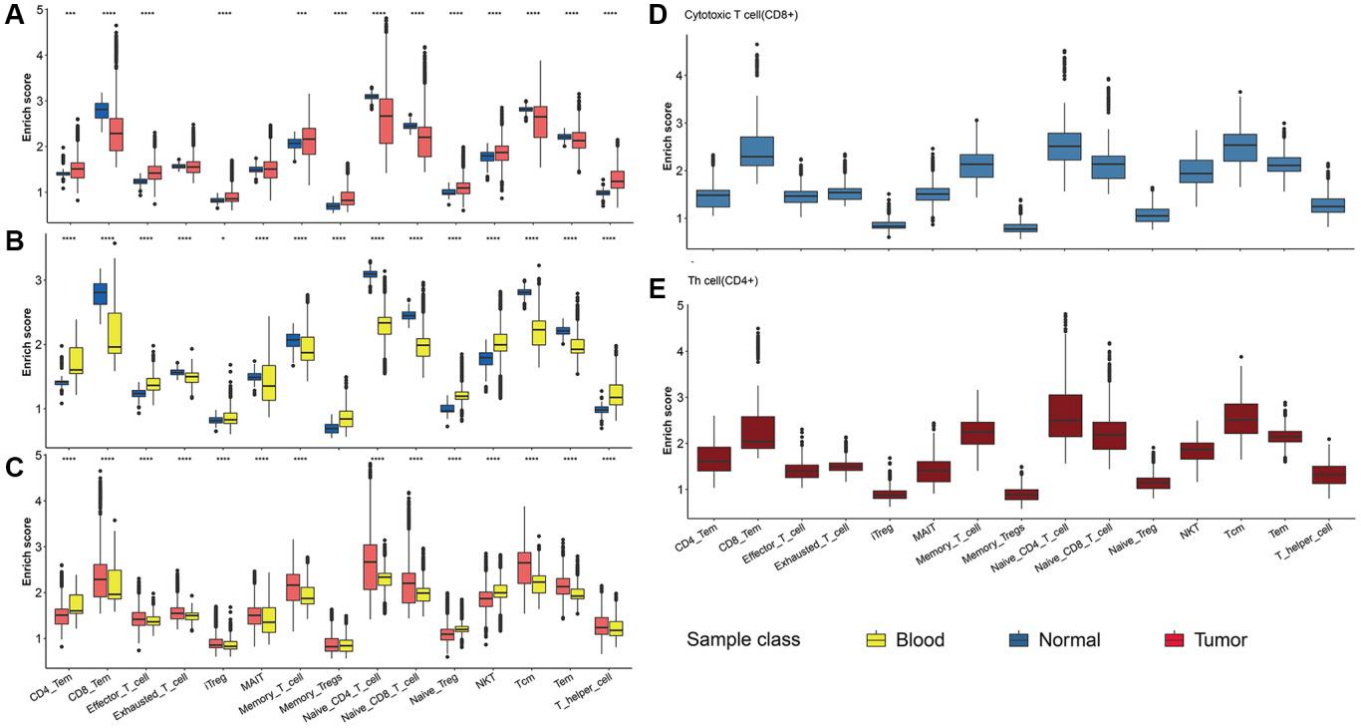
**T cell subpopulation analysis.** (**A**–**C**) The abundance of 17 T cell subpopulations in three NSCLC samples. Comparisons between two groups were calculated based on Student’s *t* test. (**D**, **E**) The abundance of CD4+ and CD8+ T cell subpopulations in 17 T cell subpopulations.

Considering the great impact of T cells on tumor progression, we focused on the T cells subpopulation with distinct phenotypes. Here we collected the maker of 17 T cell subpopulations with different phenotype functions (https://www.cellsignal.com/pathways/immune-cell-markers-human). The abundance of various T cell subpopulations in three NSCLC samples was shown in Figure 7A. Most T cells were up-regulated in tumor samples (including tissue and blood) compared with the normal group. Furthermore, we found that the distribution pattern of the 17 T cell subpopulation in CD8+ T cells was similar to CD4+ T cell, with the CD8+ T cell and naïve T cell highest infiltration (Figure 7B–7E).

### Analysis of T cell development trajectory

**Figure 8:**
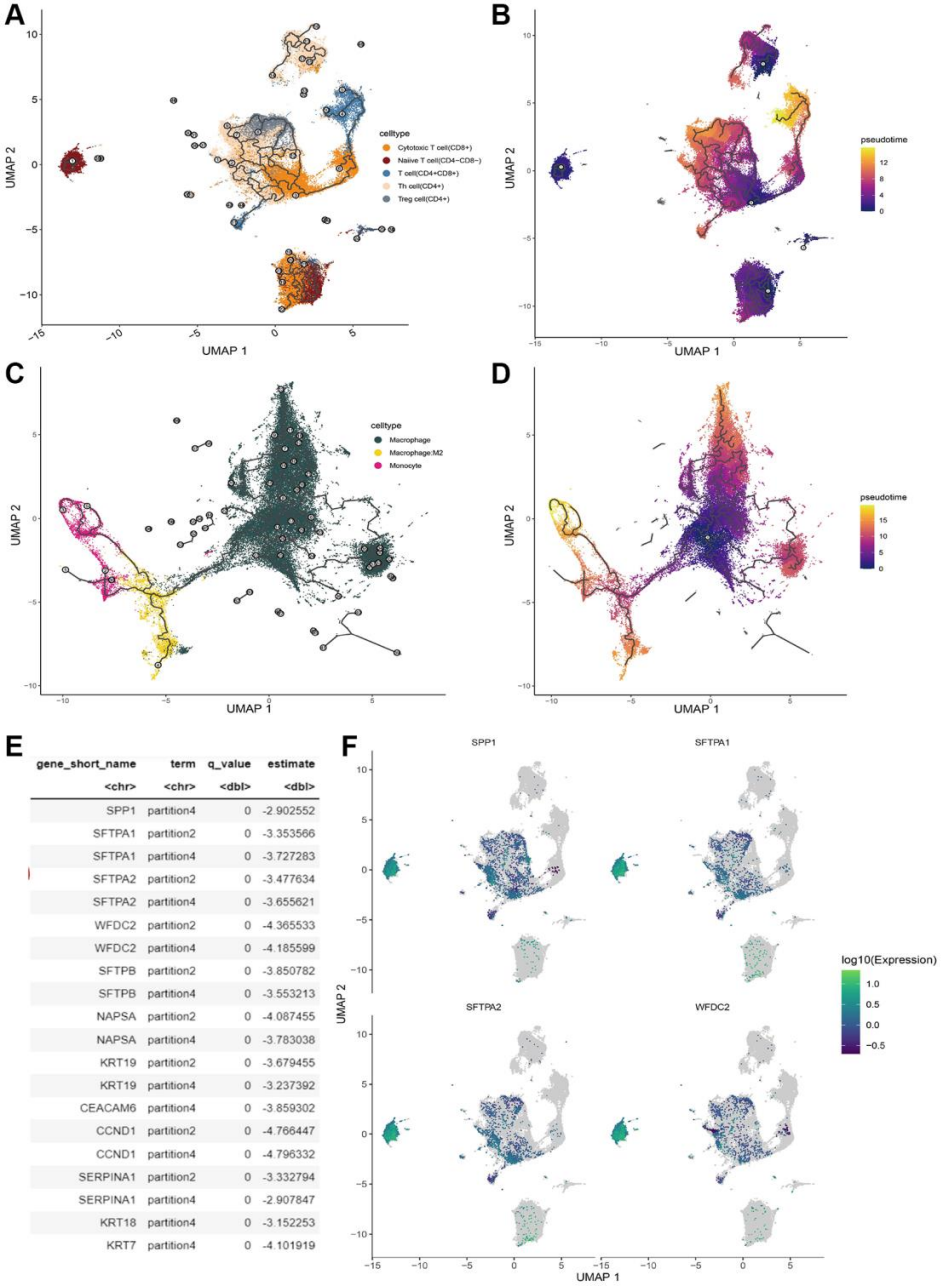
**T cell trajectory analysis.** (**A**) The evolution pathway of T cells. (**B**) The pseudo-time sequence of evolution of T cells. (**C**) The evolution pathway of macrophage. (**D**) The pseudo-time sequence of evolution of macrophages. (**E**) Significant marker gene lists involved T cell development. (**F**) Gene expression pattern of the significant marker gene in T cell.

Trajectory analysis can reshape the dynamic evaluation process of cell development and maturity by focusing on the different stage cells with distinct gene signatures. Here we dissected the trajectory development process of T cells and myeloid cells, respectively by performing correlation analysis between marker genes and developmental trajectories of T cell and myeloid cell via Monocle3. These significant genes were selected for the following trajectory analysis via Monocle3. The developmental trajectory of T cells was shown in Figure 8A, 8B. There was heterogeneity in the differentiation pathway of T cells with distinct phenotypes. Conversely, both M2-macrophage and monocyte evaluation toward macrophage (Figure 8C, 8D). In addition, we identified 57 marker genes that were found to exert vital function in the developmental trajectories of T cells (Figure 8E). We also investigated the expression pattern of their expression in T cells (Figure 8F). Most marker genes were down-regulated in naïve T cells and they were expressed in CD8+ and CD4+ T cells.

### TCR repertoire analysis

**Figure 9:**
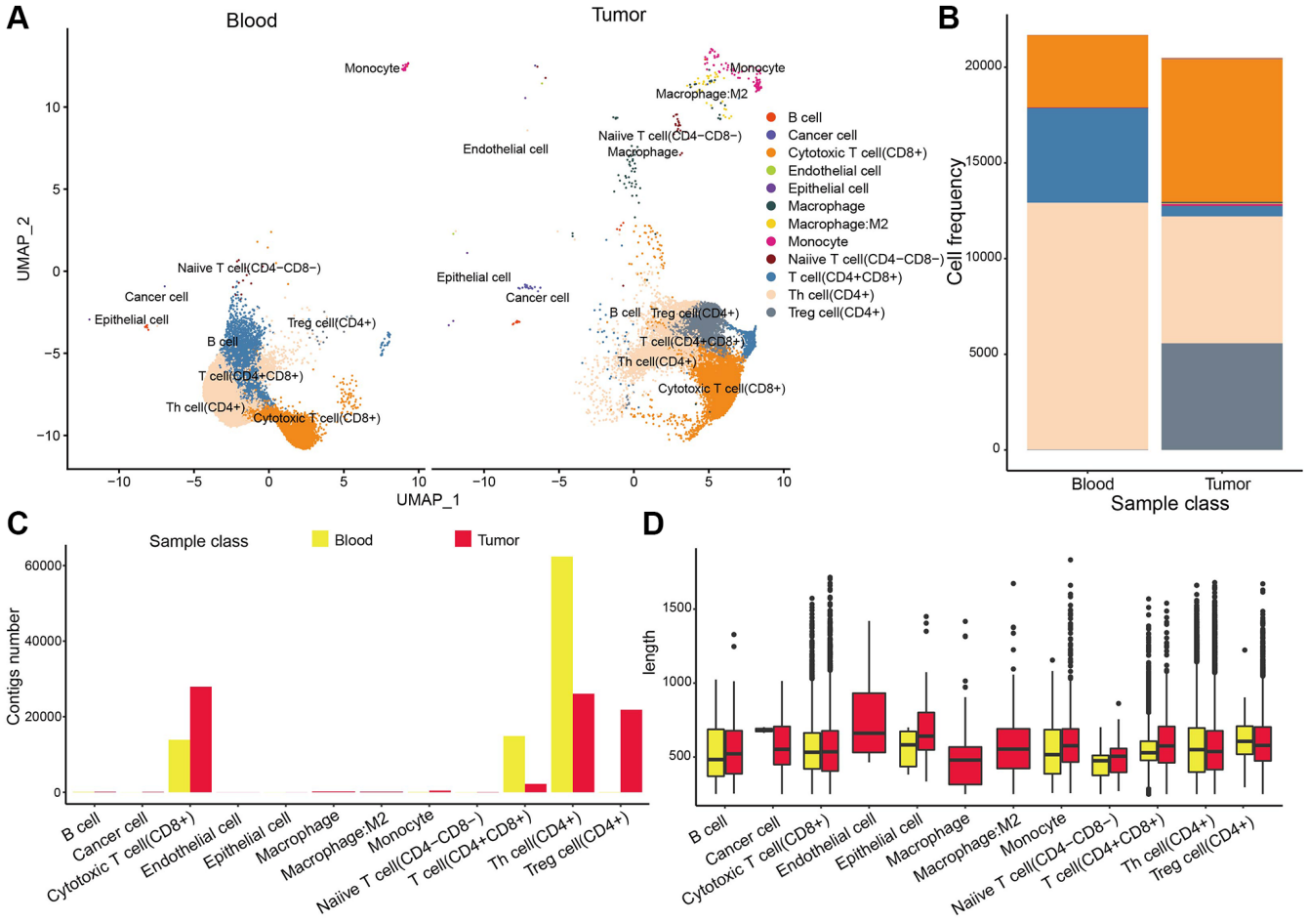
**TCR repertoire analysis.** (**A**) The distribution of TCR in different cell types. (**B**) The cell percentages of TCR in tumor samples. (**C**, **D**) TCR colony numbers and length in different types cell of tumor samples.

TCR belongs to the immunoglobulin superfamily and is a characteristic mark on the surface of T cells. TCR is a heterodimer composed of two different peptide chains, which consisted of TCR1 (γ and δ chains) and TCR2 (α and β chains). The random rearrangement of genes produces TCR polymorphism, forming a huge TCR library. The polymorphism of the TCR indicates the richness of T cell types or subpopulations. Here we downloaded TCR data of 8 NSCLC samples (blood and tissue sample) from GSE162499, including 162,839 TCRs of 42,163 cells. Results suggested that TCR distribution in the NSCLC blood sample was similar to the disposal pattern in NSCLC tumor tissue (Figure 9A, 9B). Most TCRs were identified as T cells rather than cancer cells or B cells, among which CD4+ and CD8+ T cells had the highest percentages while the CD4- and CD8- (double negative, DN T cell) carried the lowest TCRs, suggesting the maturity and function complexity of T cells (Figure 9C, 9D). In addition, we observed that the CD4+ T cell in tissues was higher than that in blood, demonstrating the great potential of tumor infiltration of CD4+ T cells. Notably, there was an additional TCR repertoire of Treg cell subpopulation in tissue, implying that Treg cells engaged in tumor microenvironment reshaping.

## DISCUSSION

The tumor ecosystem consists of multiple cell components including cancer cells, tumor-infiltrating lymphocytes, stromal cells, endothelial cells, and other supporting ingredients. The cross-talk between cell partners and their inter-communications of themselves created a dynamic and complex local microenvironment manipulating the overall tumor progression. The tumor ecosystem was also termed a tumor microenvironment (TME). More importantly, a plethora of research has manifested that plastic TME with heterogeneity is recognized as a predominant hallmark in cancer immunotherapy. For example, according to a recent study, a platelet protein, TLT-1 was found to recognize the specific CD3ε region of CD8+ T cells, impeding the anti-tumor function of CD8+ T cells by regulating the NF-κB pathway [25]. Enhancing the CD137 expression of T cells boosted the cytotoxic effector function of T cells by activating CD8+ T cells, improving the tumor OS [26]. In addition, anti- PD-1 immunotherapy manipulated CD4^+^ T cell chemotaxis response by reshaping CD11b^+^ neutrophil degranulation activity, which was considered a clinical indicator of successful pancreatic ductal adenocarcinoma (PDAC) immunotherapy [26]. Anti-cancer immunotherapy has made a great breakthrough in recent years. Of note is the need to consider the heterogeneity of tumor-infiltrating immune cells. Here, we investigated the overall cell components in the TME of NSCLC. By integrating two NSCLC-associated scRNA-seq datasets (including tumor tissue, para-tumor normal tissue, and blood samples), we identified 29 distinct cell clusters and most cell clusters were mainly enriched in the tumor tissue group. These results indicated that tumor tissue was a complex cell ecosystem, implying the existence of heterogeneity. Because of the high proportion of cluster 0, we obtained significant cluster-specific genes in cluster 0. They were chiefly involved in T cell activation and autoimmune disease-associated pathways.

Furthermore, 285 cancer cell marker genes, 142 T cell marker genes, and 225 macrophage marker genes were identified by taking the intersection between DEGs (tumor vs. normal) and immune cell marker genesets. 17 marker genes were associated with NSCLC survival. KRT6A has been reported to be a negative regulator in NSCLC survival [27], which was in agreement with our analysis. An isolated study based on transcriptome data also validated the efficacy of ADM as an independent prognosis factor [28]. Here we exemplified that ADM was a key clinical factor in evaluating NSCLC survival at the single-cell level. Enrichment analysis indicated that T and B cell marker genes principally engaged in response to interferon-gamma (IFN-γ), which was indispensable to the cancer-killing of CAR-T cell therapy. A recent study suggested that the knockout of genes in the IFN γ receptor signal pathway (IFNGR1, JAK1, or JAK2) endowed solid tumor cells with resistance to CAR-T cells by reducing the overall binding time and affinity of CAR-T cells with the cancer cells [29]. Macrophage marker genes were associated with neutrophil activation. Within the tumor background, neutrophils derive bone marrow-derived suppressor cell (MDSCs) subsets, which suppressed the anti-tumor response of T cells by regulating PD-L1 expression to promote angiogenesis [30, 31]. The cancer cell marker gene was significantly enriched in xenobiotic metabolism. The metabolic preferences acquired by cancer cells were a favorable factor in the nutritional requirements of cancer cell growth and proliferation, validating the profound influence of cell metabolism reprogramming on tumorigenesis [32]. The unique metabolic characteristic of cancer cells endowed them with the ability to obtain necessary nutrients from a nutrient-deficient environment, which was vital to maintain viability and generating new biomass [33].

As previously described, T cells subpopulations with distinct phenotypes varied in anti-tumor activity. Herein, 17 T cell subpopulations were enrolled in our analysis. naïve T (DN T cell) cell was identified as the highest infiltration T cell subpopulations in tumor tissue and blood samples. It is widely accepted that CD3+CD4-CD8- DNT cells are a distinct subset of T cells. DNTs carried an effective cancer-killing potential in NSCLC models. The expanding DNTs *in vivo* acquired an anti-tumor phenotype with the NK cell marker NKG2D, DNAM-1, which mediated the cytotoxicity response targeting cancer cells by secreting cytotoxic cytokines such as IFNγ and soluble TRAIL (sTRAIL) [34]. Emerging evidence validated that TCRαβ+ DNTs and TCRγδ+ DNTs displayed equivalent anti-tumor activity in a series of preclinical models. There was the DNT cell subpopulation in tumor-infiltrating lymphocytes derived NSCLC samples, together with increased expression of PD-1. Interestingly, the DNT cells extracted from healthy donors curtailed late-stage lung cancer progression through which anti-PD-1 therapy contributed to DNT cell infiltration into primary tumor biomass [35]. These results, combined with our findings, manifested that targeting DNT cells could be a novel platform in immunotherapy.

The cell trajectory analysis based on the correlation between cell marker genes and trajectories-associated genes via Monocle3 indicated the temporal heterogeneity of NSCLC-infiltrating T cells. We found that 57 marker genes were associated with the T cells maturation process. Most marker genes were down-regulated in naïve T cells (considered as immature T cells) and expressed in mature T cells such as CD8+ and CD4+ T cells. These results demonstrated that 57 marker genes were important driving forces in T cell development. Consistent with this point, our TCR analysis indicated that the TCR numbers of early-stage T cells were lower than the mature-stage T cells, including CD4+ and CD8+ T cells. These data showed that the dynamic changes of the TCR repertoire library could be a sensitive indicator of the T cell development process. In addition, our data revealed an additional TCR repertoire of Treg cell subpopulation in tumor tissue.

From a clinical perspective, understanding the behavior and molecular signatures of Treg cells in the TME is paramount. While some studies have demonstrated the immune-suppressive attributes of Treg cells in the TME, which aids tumor growth and immune evasion [36, 37]. However, another study discovered Treg cells restrained tumor growth and increased response to immunotherapy by up taking lactic acid [38]. This dichotomy suggests a nuanced metabolic balance within Treg cells. Clinically, manipulating this balance could offer a dual therapeutic approach: impairing tumor growth and promoting immune response by reshaping the metabolic landscape within the TME.

Given this data, it becomes clear that understanding the intricacies of T cell trajectory and the TCR repertoire can provide critical insights for enhancing patient care. Harnessing this point can guide the development of personalized therapeutic strategies for NSCLC, thereby improving patient outcomes and response rates to treatments.

## CONCLUSION

Taken together, our data revealed the heterogeneity of TME, the T with different phenotypes cell of TME provides a theoretical basis for variable immunotherapy response.

## Supplementary Materials

Supplementary File 1

Supplementary Table 1
