# Supplementary material for: Metabolic and senescence characteristics associated with the immune microenvironment in non-small cell lung cancer: insights from single-cell RNA sequencing
Source: Aging (Albany NY). 2023 Oct 26;15(20):11571–87. doi: 10.18632/aging.205146 (PMC10637824; doi:10.18632/aging.205146)
Supplement: Supplementary File 1 [file aging-15-205146-s001.docx]

**Supplementary File 1. R code script of the bioinformatical analysis.**

library(ggunchained)

library(ggplot2)

library(ggsignif)

library(tidyverse)

library(ggpubr)

library(Seurat)

#Figure1

projectID <- "GSE117570"

cancer <- "NSCLC"

GSE117570_sample_list <- c("GSM3304007_P1_Tumor","GSM3304008_P1_Normal","GSM3304009_P2_Tumor","GSM3304010_P2_Normal",

"GSM3304011_P3_Tumor","GSM3304012_P3_Normal","GSM3304013_P4_Tumor","GSM3304014_P4_Normal")

for(i in 1:8){

sample = GSE117570_sample_list[i]

mtx_file <- paste0(analysis_path,"/1.data/GSE117570/",sample,"_processed_data.txt.gz")

mtx <- read.table(mtx_file)

sobj <- CreateSeuratObject(counts = mtx, min.features = 100, min.cells = 10, project = sample)

sobj[["percent.mito"]] <- PercentageFeatureSet(sobj, pattern = "^MT-")

VlnPlot(sobj, features = c("nFeature_RNA", "nCount_RNA", "percent.mito"), ncol = 3)->p

p_file <- paste0(analysis_path,"/2.qc/GSE117570/",sample,".QC.pdf")

ggsave(p_file,p)

sobj <- subset(sobj, subset = nFeature_RNA > 100 & nFeature_RNA < 5000 & percent.mito < 20 & nCount_RNA > 10)

assign(paste0("GSE117570_sobj",i),sobj)

}

sobj_list <- c(GSE117570_sobj1,GSE117570_sobj2,GSE117570_sobj3,GSE117570_sobj4,GSE117570_sobj5,GSE117570_sobj6,GSE117570_sobj7,GSE117570_sobj8)

GSE117570_merge_sobj <- merge(x = GSE117570_sobj1,

y = c(GSE117570_sobj2,GSE117570_sobj3,GSE117570_sobj4,GSE117570_sobj5,GSE117570_sobj6,GSE117570_sobj7,GSE117570_sobj8))

#GSE162498

cancer <- "NSCLC"

projectID <- "GSE162498"

sample_dir_list <- c("P34_Tumor_raw","P35_Tumor_raw","P42_Tumor_raw","P43_Tumor_raw","P46_Tumor_raw","P47_Tumor_raw","P55_Tumor_raw",

"P57_Blood_raw","P57_Tumor_raw","P58_Blood_filtered","P58_Tumor_filtered","P60_Blood_filtered","P60_Juxta_ffiltered",

"P60_Tumor_filtered","P61_Blood_filtered","P61_Juxta_filtered","P61_Tumor_filtered")

sample_list <- c("P34_Tumor","P35_Tumor","P42_Tumor","P43_Tumor","P46_Tumor","P47_Tumor","P55_Tumor",

"P57_Blood","P57_Tumor","P58_Blood","P58_Tumor","P60_Blood","P60_Juxta",

"P60_Tumor","P61_Blood","P61_Juxta","P61_Tumor")

for(i in 1:17){

print(i)

mtx_file <- paste0(analysis_path,"/1.data/GSE162498/",sample_dir_list[i],"_feature_bc_matrix")

mtx <- Read10X(mtx_file)

sobj <- CreateSeuratObject(counts = mtx, min.features = 100, min.cells = 10, project = sample_list[i])

sobj[["percent.mito"]] <- PercentageFeatureSet(sobj, pattern = "^MT-")

VlnPlot(sobj, features = c("nFeature_RNA", "nCount_RNA", "percent.mito"), ncol = 3)->p

p_file <- paste0(analysis_path,"/2.qc/",projectID,"/",sample_list[i],".QC.pdf")

ggsave(p_file,p)

sobj <- subset(sobj, subset = nFeature_RNA > 100 & nFeature_RNA < 5000 & percent.mito < 20 & nCount_RNA > 10)

assign(paste0("Sobj",i),sobj)

}

sobj_list <- c( Sobj1,Sobj2, Sobj3, Sobj4, Sobj5, Sobj6, Sobj7, Sobj8, Sobj9, Sobj10, Sobj11, Sobj12, Sobj13, Sobj14, Sobj15,

Sobj16, Sobj17)

GSE162498_merge_sobj <- merge(x = Sobj1,y = c(Sobj2, Sobj3, Sobj4, Sobj5, Sobj6, Sobj7, Sobj8, Sobj9, Sobj10, Sobj11, Sobj12, Sobj13, Sobj14, Sobj15,

Sobj16, Sobj17))

#remove batch

cancer <- "NSCLC"

project_list <- c("GSE117570","GSE162498")

P_rds_1 <- GSE117570_merge_sobj

P_rds_2 <- GSE162498_merge_sobj

sobj_list <- c(P_rds_1,P_rds_2)

sobj_list <- lapply(X = sobj_list, FUN = function(x) {

x <- NormalizeData(x)

x <- FindVariableFeatures(x, selection.method = "vst", nfeatures = 2000)

})

features <- SelectIntegrationFeatures(object.list = sobj_list)

sobj_anchors <- FindIntegrationAnchors(object.list = sobj_list, anchor.features = features)

sobj_combined <- IntegrateData(anchorset = sobj_anchors)

rds <- sobj_combined

use.genes <- rds@assays$integrated@var.features

rds <- ScaleData(rds, features = use.genes)

rds <- RunPCA(object = rds, features = use.genes, do.print = FALSE)

rds <- FindClusters(rds, resolution = 0.6)

rds = RunUMAP(rds, dims=1:20)

rds <- RunTSNE(rds, dims = 1:20, do.fast = TRUE, check_duplicates = FALSE)

#Figure1.A,B,C

DimPlot(rds, reduction = "umap",label = TRUE)->p

p_file <- paste0(analysis_path,"/3.cluster/umap.cluster.pdf")

ggsave(p_file,p,width = 8)

DimPlot(rds, reduction = "umap",group.by = "sample")->p

p_file <- paste0(analysis_path,"/3.cluster/umap.sample.pdf")

ggsave(p_file,p,width = 8)

DimPlot(rds, reduction = "umap",group.by = "sample",split.by = "sample")->p

p_file <- paste0(analysis_path,"/3.cluster/umap.sample.split.pdf")

ggsave(p_file,p,width = 8)

#Celltype annotation(see Method)

nsclc_celltype <- list('0' = "Macrophage" ,

'1' = "Th cell(CD4+)",

'2' = "Cytotoxic T cell(CD8+)",

'3' = "Cytotoxic T cell(CD8+)",

'4' = "Th cell(CD4+)",

'5' = "T cell(CD4+CD8+)",

'6' = "Treg cell(CD4+)",

'7' = "Th cell(CD4+)",

'8' = "Naiive T cell(CD4-CD8-)",

'9' = "Macrophage",

'10' = "Cytotoxic T cell(CD8+)",

'11' = "Naiive T cell(CD4-CD8-)",

'12' = "Th cell(CD4+)",

'13' = "Macrophage:M2",

'14' = "Macrophage",

'15' = "Monocyte",

'16' = "T cell(CD4+CD8+)",

'17' = "Monocyte",

'18' = "Cancer cell",

'19' = "Epithelial cell",

'20' = "Cancer cell",

'21' = "B cell",

'22' = "Cancer cell",

'23' = "Epithelial cell",

'24' = "Macrophage",

'25' = "Endothelial cell",

'26' = "Epithelial cell",

'27' = "Epithelial cell",

'28' = "Naiive T cell(CD4-CD8-)")

rds_file <- "5.celltype_annotation/celltype.rds"

rds = readRDS(rds_file)

#Figure1.D

celltype_color = c("DarkOrange","GreenYellow","Purple","DarkSlateGray","Gold","DeepPink2","Red4","#4682B4",

"#FFDAB9","#708090","#836FFF","#CDC673","#CD9B1D","#FF6EB4","#CDB5CD","DarkGreen")

names(celltype_color)<-unique(rds$celltype)

DimPlot(rds,group.by = "celltype",cols = celltype_color,label = TRUE, repel =TRUE)->p

p_file <- paste0("5.celltype_annotation/celltype.umap.pdf")

ggsave(p_file,p,width = 8.5)

#Figure1.E,F,G

ggplot(rds@meta_data,aes(x = sample_class,fill = celltype))+

geom_bar(width = 0.8, position = "fill")+

theme_classic()+

scale_fill_manual(values = celltype_color)+

theme(

axis.text.x=element_text(color = "black", size=15,angle = 30,hjust =1),

axis.text.y=element_text(color = "black",size=15),

axis.title.x=element_text(color = "black", size = 15),

axis.title.y=element_text(color = "black", size = 15),

legend.text = element_text(color = "black", size=15))->p.f1.E

ggplot(rds@meta_data%>%filter(celltype %in% c("Cytotoxic T cell(CD8+)","Th cell(CD4+)",

"T cell(CD4+CD8+)","Treg cell(CD4+)","Naiive T cell(CD4-CD8-)")),

aes(x = sample_class,fill = celltype))+

geom_bar(width = 0.8, position = "fill")+

theme_classic()+

scale_fill_manual(values = celltype_color)+

theme(

axis.text.x=element_text(color = "black", size=15,angle = 30,hjust =1),

axis.text.y=element_text(color = "black",size=15),

axis.title.x=element_text(color = "black", size = 15),

axis.title.y=element_text(color = "black", size = 15),

legend.text = element_text(color = "black", size=15))->p.f1.F

ggplot(rds@meta_data%>%filter(celltype %in% c("Macrophage:M2","Macrophage","Monocyte")),

aes(x = sample_class,fill = celltype))+

geom_bar(width = 0.8, position = "fill")+

theme_classic()+

scale_fill_manual(values = celltype_color)+

theme(

axis.text.x=element_text(color = "black", size=15,angle = 30,hjust =1),

axis.text.y=element_text(color = "black",size=15),

axis.title.x=element_text(color = "black", size = 15),

axis.title.y=element_text(color = "black", size = 15),

legend.text = element_text(color = "black", size=15))->p.f1.G

#Figure2

rds_file <- "5.celltype_annotation/celltype.rds"

rds = readRDS(rds_file)

marker_cluster = FindAllMarkers(rds)

marker_cluster%>%filter(p.val<0.05)%>%

group_by(cluster)%>%

top_n(10,wt=avg_log2FC)->top10_degs

DoHeatmap(rds,features = unique(top10_degs$gene))

for(gene in unique(top10_degs$gene)){

FeaturePlot(rds,features = gene)->p

p_file = paste0("5.celltype_annotation/fp.",gene,".pdf")

ggsave(p_file,p)

}

library(clusterProfiler)

library(org.Hs.eg.db)

GO_enrich <- function(genelist,cluster){

eg <- bitr(genelist,

fromType="SYMBOL",

toType=c("ENTREZID","ENSEMBL",'SYMBOL'),

OrgDb="org.Hs.eg.db")

go <- enrichGO(eg$ENTREZID,

OrgDb = org.Hs.eg.db,

ont='ALL',

pAdjustMethod = 'BH',

pvalueCutoff = 0.05,

qvalueCutoff = 0.05,

keyType = 'ENTREZID',

readable = TRUE)

write.table(go,,filename = paste0("go.",cluster,".csv"), sep=",")

dotplot(go,showCategory=25,orderBy = "p.adjust")->p

ggsave(paste0("go.",cluster,".pdf"),p)

return(go)

}

KEGG_enrich <- function(genelist,cluster){

eg <- bitr(genelist,

fromType="SYMBOL",

toType=c("ENTREZID","ENSEMBL",'SYMBOL'),

OrgDb="org.Hs.eg.db")

kegg <- enrichKEGG(eg$ENTREZID,

organism = 'hsa',

keyType = 'kegg',

pvalueCutoff = 0.05,

pAdjustMethod = 'BH',

minGSSize = 3,

maxGSSize = 500,

qvalueCutoff = 0.05,

use_internal_data = FALSE)

kegg <- setReadable(kegg, OrgDb = org.Hs.eg.db, keyType="ENTREZID")

write.table(kegg,,filename = paste0("kegg.",cluster,".csv"), sep=",")

barplot(kegg,showCategory=25,orderBy = "p.adjust")->p

ggsave(paste0("kegg.",cluster,".pdf"),p)

return(kegg)

}

marker_cluster%>%filter(p.val<0.05)->marker_cluster_flt

for(clu in unique(marker_cluster_flt$cluster)){

marker_cluster_flt%>%filter(cluster==clu)->tmp_df

genelist = unique(tmp_df$gene)

GO_enrich(genelist,clu)

KEGG_enrich(genelist,clu)

}

#Figrue3

rds_file <- "5.celltype_annotation/celltype.rds"

rds = readRDS(rds_file)

Idents(rds) <- "celltype"

FindAllmarkers(rds)->marker_celltype

for(ct in unique(marker_celltype$cluster)){

subset(rds, celltype == ct) -> rds_ct

# Normal Vs Tumor

diff_dat_N_T <- FindMarkers(rds_ct,ident.1="Normal",ident.2="Tumor",

group.by='sample_class')

colnames(diff_dat_N_T) <- paste0(colnames(diff_dat_N_T),"_N_T")

diff_dat_N_T%>%

mutate(gene = rownames(diff_dat_N_T))%>%

filter(p_val_adj_N_T < 0.001)->diff_dat_N_T

diff_dat_N_T%>%

select(avg_log2FC_N_T,p_val_adj_N_T,gene)%>%

mutate(Group = "N.vs.T")->diff_dat_N_T_1

colnames(diff_dat_N_T_1) <- c("log2FC","P.val.adj","Gene","Group")

# Normal Vs PBMC

diff_dat_N_B <- FindMarkers(rds_ct,ident.1="Normal",ident.2="Blood",

group.by='sample_class')

colnames(diff_dat_N_B) <- paste0(colnames(diff_dat_N_B),"_N_B")

diff_dat_N_B%>%

mutate(gene = rownames(diff_dat_N_B))%>%

filter(p_val_adj_N_B < 0.001)->diff_dat_N_B

diff_dat_N_B%>%

select(avg_log2FC_N_B,p_val_adj_N_B,gene)%>%

mutate(Group = "N.vs.B")->diff_dat_N_B_1

colnames(diff_dat_N_P_1) <- c("log2FC","P.val.adj","Gene","Group")

#Tumor Vs Blood

diff_dat_T_B <- FindMarkers(rds_ct,ident.1="Tumor",ident.2="Blood",

group.by='sample_class')

colnames(diff_dat_T_B) <- paste0(colnames(diff_dat_T_B),"_T_B")

diff_dat_T_B%>%

mutate(gene = rownames(diff_dat_T_B))%>%

filter(p_val_adj_T_B < 0.001)->diff_dat_T_B

diff_dat_T_B%>%

select(avg_log2FC_T_B,p_val_adj_T_B,gene)%>%

mutate(Group = "T.vs.B")->diff_dat_T_B_1

colnames(diff_dat_T_B_1) <- c("log2FC","P.val.adj","Gene","Group")

diff_data<-rbind(diff_dat_N_T_1,diff_dat_N_B_1,diff_dat_T_B_1)

marker_celltype%>%filter(cluster==ct,p.val<0.05)->marker_celltype_spe

diff_data%>%filter(Gene %in% marker_celltype_sep$gene)->diff_data_spe

write.table(diff_data_spe,"filename")

ggplot(diff_data_spe,aes(x = Group,y = Gene))+

geom_point(aes(fill = log2FC,size = -log10(`P.val.adj`)),shape = 21,alpha = 0.8)+

scale_fill_continuous( high=clustcol[17])+

theme_classic()+

theme(

axis.text.x=element_text(color = "black", size=12),

axis.text.y=element_text(color = "black", size=7),

axis.title.x=element_text(color = "black", size = 13,face = "bold"),

axis.title.y=element_text(color = "black", size = 13,face = "bold")

)->p

p_file <- paste0("5.celltype_annotation/marker_diff_exp/",ct,".pdf")

ggsave(p_file,p,height = 8,width = 6.5)

}

#Survival analysis

library(survminer)

#exp = readr::read_tsv("reference/TCGA/TCGA-LUAD.htseq_fpkm.tsv.gz")

#sur_df = readr::read_tsv("reference/TCGA/TCGA-LUAD.survival.tsv")

exp = readr::read_tsv("reference/TCGA/TCGA-LUSC.htseq_counts.tsv.gz")

sur_df = readr::read_tsv("reference/TCGA/TCGA-LUSC.survival.tsv")

k=keys(org.Hs.eg.db,keytype = "ENSEMBL")

glist=select(org.Hs.eg.db,keys=k,columns = c("ENTREZID","SYMBOL"), keytype="ENSEMBL")

data.frame(gene=character(),pvalue = double(),celltype = character(),group = character())->pvalue_df

for(ct in unique(rds$celltype)){

diff_dat_spe_file <- paste0("5.celltype_annotation/marker_diff_exp/",ct,".diff.spe.csv")

diff_dat_spe <- readr::read_csv(diff_dat_spe_file)

for(i in 1:nrow(diff_dat_spe)){

gene = diff_dat_spe$gene_symbol[i]

group = diff_dat_spe$group[i]

print(gene)

glist%>%

filter(SYMBOL == gene)-> gene_trans

gene_trans[order(gene_trans$ENSEMBL),]->gene_trans

if(nrow(gene_trans)>0){

for(ensembl in gene_trans$ENSEMBL){

data.frame(t(exp[grepl(ensembl,exp$Ensembl_ID),-1]))->gene_exp

if(ncol(gene_exp)>0){break}

}

colnames(gene_exp) <- c("exp")

if(nrow(gene_exp)>1&sum(gene_exp$exp)>0){

data.frame(gene_exp)%>%mutate(sample = rownames(gene_exp))->gene_exp

sur_df%>%

left_join(gene_exp)->sur_df_exp

#print(head(sur_df_exp))

sur_df_exp[is.na(sur_df_exp)] <- 0

sur_df_exp%>%

group_by(OS,`_PATIENT`,`OS.time`)%>%

summarize(exp_fit = mean(exp))%>%

ungroup()%>%

mutate(group = ifelse(exp_fit > median(exp_fit),"High","Low"))%>%

dplyr::select(-exp_fit)->sur_df_exp

colnames(sur_df_exp) <- c("Status","Patient","Time","Group")

fit_1 <- survfit(Surv(Time, Status) ~ Group, #group factor: sex

data = sur_df_exp)

#print(head(sur_df_exp))

#print(fit)

surv_pvalue(fit_1)->sp

data.frame(gene = gene, pvalue = sp$pval,celltype = ct,group = group)->tmp_df

pvalue_df <- rbind(pvalue_df,tmp_df)

#print(pvalue_df)

ggsurvplot(fit_1,

pval = TRUE,

conf.int = TRUE,

risk.table = TRUE, # Add risk table

risk.table.col = "Group", # Change risk table color by groups

#linetype = "Group", # Change line type by groups

surv.median.line = "hv", # Specify median survival

ggtheme = theme_bw(), # Change ggplot2 theme

palette = c("#E7B800", "#2E9FDF"))->p

pdf(paste0("08.survival.features/LUSC/",ct,".",gene,".pdf"))

print(p)

dev.off()

}else{print("no exp")}

}else{print("No gene found")}

}

}

#Figure4

pvalue_df%>%filter(pvalue<0.05)-pamg_df

for(gene in pamg_df$gene){

FeaturePlot(rds,feature = gene)->p

p_file = paste0("6.marker.diff.spe/fp.",gene,".pdf")

ggsave(p_file,p)

}

DoHeatmap(rds,features = pamg_df$gene,gruop.by = "celltype")

#plot KRT6A,ADM,NAPSA

VlnPlot(rds,featrues = "KRT6A",group.by = "celltype",split.by = "sample_class")

rds[["KRT6A"]]<-rds@assays$RNA@counts["KRT6A",]

options(repr.plot.height=4 , repr.plot.width=12)

ggplot(gene_meta%>%filter(sample_class %in% c("Blood","Normal"),KRT6A>0),aes(x = celltype,y = log2(KRT6A+1),fill = sample_class))+

geom_split_violin()+

theme_classic()+

scale_fill_manual(values = list("Normal"="#0066CC","Blood"="#FFFF00"))+ #"Tumor" = "#FF6666","Blood"="#FFFF00","Normal"="#0066CC"

ylab("")+

xlab("")+

stat_compare_means(aes(group = sample_class),label = "p.signif",hide.ns = TRUE)+

theme(

axis.text.y = element_text(color = "black", size=15), #不显示坐标刻度

#axis.ticks.y = element_text(color = "black", size=15),

axis.title = element_text(color = "black", size=13),

axis.text.x = element_text(color = 'black',size = 13,angle = 30,hjust =1,vjust = 1),

legend.title = element_text(color = "black", size=13),

legend.text = element_text(color = "black", size=13)

)->p

p

#legend.position = 'none')

p_file = paste0(analysis_path,"/vln.B.N.KRT6A.gt0.pdf")

ggsave(p_file,p,width = 11,height = 4.5)

#Figure5

marker_celltype%>%filter(p.val<0.05)->marker_celltype_flt

for(clu in unique(marker_celltype_flt$cluster)){

marker_cluster_flt%>%filter(cluster==clu)->tmp_df

genelist = unique(tmp_df$gene)

GO_enrich(genelist,clu)

KEGG_enrich(genelist,clu)

}

#Figrue6

library(stringr)

library(tidyverse)

library(enrichplot)

hall_gmt<-read.gmt("/Personal/fuxin/dfuxin/Bioinfo_Scrs/scRNA_biological_analysis/reference/MigDB/h.all.v7.5.1.entrez.gmt")

immun_gmt <- read.gmt("/Personal/fuxin/dfuxin/Bioinfo_Scrs/scRNA_biological_analysis/reference/MigDB/c7.all.v7.5.1.entrez.gmt")

rds_file <- "5.celltype_annotation/celltype.rds"

obj <- readRDS(rds_file)

diff_dat_sum = readr::read_csv("6.marker_diff_spe/diff_dat_spe_sum.csv")

for(ct in unique(rds$celltype)){

diff_dat_sum%>%

filter(celltype== ct) -> dds_tmp

genelist = unique(dds_tmp$Gene)

genelist = bitr(genelist,fromType="SYMBOL",toType="ENTREZID",OrgDb="org.Hs.eg.db")

genelist <- dplyr::distinct(genelist,SYMBOL,.keep_all=TRUE)

genelist%>%

left_join(dds_tmp, by = c("SYMBOL" = "Gene"))->genelist

genelist[order(genelist$log2FC,decreasing = TRUE),]->genelist

geneList <- genelist$log2FC

names(geneList) <- genelist$ENTREZID

hall_res<-GSEA(geneList,TERM2GENE = hall_gmt)

immun_res <- GSEA(geneList,TERM2GENE = immun_gmt)

if(nrow(immun_res)>0){

if(nrow(immun_res)==1){

write.table(immun_res,paste0(gsea_dir,"/",ct,".immun.csv"))

gseaplot2(immun_res, geneSetID = 1, title = immun_res$Description[1])->p

p_file <- paste0(gsea_dir,"/",ct,".immun.gsea.pdf")

ggsave(p_file,p,width = 6,height = 5)

}else{

write.table(immun_res,paste0(gsea_dir,"/",ct,".immun.csv"))

data.frame(immun_res)->immun_res_df

immun_res_df[order(immun_res_df$p.adjust),]->immun_res_df

ggplot(immun_res_df[1:20,], aes(NES, fct_reorder(Description, NES), fill=qvalues)) +

geom_bar(stat='identity',width = 0.8) +

scale_fill_continuous(high='#99CC66', low='#FF6666', guide=guide_colorbar(reverse=TRUE)) +

theme_bw() +

theme(

axis.text.x=element_text(color = "black", size=13),

axis.text.y=element_text(color = "black", size=13),

axis.title.x=element_text(color = "black", size = 13 ,face = "bold"),

axis.title.y=element_text(color = "black", size = 13,face = "bold")

)+ylab(NULL)->p

p_file <- paste0(gsea_dir,"/",ct,".immun.bar.pdf")

ggsave(p_file,p, width = 16,height = 8)

gseaplot2(immun_res, geneSetID = 1,title = immun_res$Description[1])->p

p_file <- paste0(gsea_dir,"/",ct,".immun.gsea.pdf")

ggsave(p_file,p,width = 6,height = 5)

dotplot(immun_res,split=".sign")+facet_grid(~.sign)->p

p_file <- paste0(gsea_dir,"/",ct,".immun.dot.pdf")

ggsave(p_file,p, width = 16,height = 8)

}

}

if(nrow(hall_res)>0){

if(nrow(hall_res)==1){

write.table(immun_res,paste0(gsea_dir,"/",ct,".hall.csv"))

gseaplot2(hall_res, geneSetID = 1, title = hall_res$Description[1])->p

p_file <- paste0(gsea_dir,"/",ct,".hall.gsea.pdf")

ggsave(p_file,p,width = 10,height = 5)

}else{

write.table(hall_res,paste0(gsea_dir,"/",ct,".hall.csv"))

data.frame(hall_res)->hall_res_df

hall_res_df[order(hall_res_df$p.adjust),]->hall_res_df

ggplot(hall_res_df[1:20,], aes(NES, fct_reorder(Description, NES), fill=qvalues)) +

geom_bar(stat='identity',width = 0.8) +

scale_fill_continuous(high='#99CC66', low='#FF6666', guide=guide_colorbar(reverse=TRUE)) +

theme_bw() +

theme(

axis.text.x=element_text(color = "black", size=12),

axis.text.y=element_text(color = "black", size=12),

axis.title.x=element_text(color = "black", size = 13,face = "bold"),

axis.title.y=element_text(color = "black", size = 13,face = "bold")

)+ylab(NULL)->p

p_file <- paste0(gsea_dir,"/",ct,".hall.bar.pdf")

ggsave(p_file,p, width = 16,height = 8)

gseaplot2(hall_res, geneSetID = 1,title = hall_res$Description[1])->p

p_file <- paste0(gsea_dir,"/",ct,".hall.gsea.pdf")

ggsave(p_file,p,width = 8,height = 8)

dotplot(hall_res,split=".sign")+facet_grid(~.sign)->p

p_file <- paste0(gsea_dir,"/",ct,".hall.dot.pdf")

ggsave(p_file,p, width = 16,height = 8)

}

}

#Figrue7

library(SingleCellExperiment)

library(Seurat)

library(GSEABase)

library(escape)

library(reshape2)

#Collected T cell marker from public database

immune_cell_marker

glist <- GeneSetCollection(immune_cell_marker))

es_score <- enrichIt(obj = rds, gene.sets = glist, groups = 1000, cores = 5)

cbind(rds@meta.data,es_score)->es_score

melt(es_score)->es_score_melt

es_score_melt$sample_class <- factor(es_score_melt$sample_class, levels = c("Normal","Tumor","Blood"))

ggplot(es_score_melt%>%filter(sample_class %in% c("Blood","Tumor")),aes(x = variable,y = value,fill = sample_class))+

geom_boxplot()+

scale_fill_manual(values = list("Blood"="#FFFF00","Tumor" = "#FF6666"))+ #"Tumor" = "#FF6666","Blood"="#FFFF00","Normal"="#0066CC"

stat_compare_means(label = "p.signif",hide.ns = TRUE)+

theme_classic()+

theme(

axis.text.y = element_text(color = "black", size=15), #不显示坐标刻度

#axis.ticks.y = element_text(color = "black", size=15),

axis.title = element_text(color = "black", size=13),

axis.text.x = element_text(color = 'black',size = 13,angle = 30,hjust =1,vjust = 1),

legend.title = element_text(color = "black", size=13),

legend.text = element_text(color = "black", size=13)

)->p

p

p_file = paste0(analysis_path,"/es_score.all.B.T.pdf")

ggsave(p_file,p,width = 12,height =5)

ggplot(es_score_melt%>%filter(celltype == "Th cell(CD4+)"),aes(x = variable,y = value))+

geom_boxplot(fill = "Red4")+

scale_fill_manual(values = list("Blood"="#FFFF00","Tumor" = "#FF6666"))+ #"Tumor" = "#FF6666","Blood"="#FFFF00","Normal"="#0066CC"

stat_compare_means(label = "p.signif",hide.ns = TRUE,ref.group = ".all.")+

theme_classic()+

theme(

axis.text.y = element_text(color = "black", size=15), #不显示坐标刻度

#axis.ticks.y = element_text(color = "black", size=15),

axis.title = element_text(color = "black", size=13),

axis.text.x = element_text(color = 'black',size = 13,angle = 30,hjust =1,vjust = 1),

legend.title = element_text(color = "black", size=13),

legend.text = element_text(color = "black", size=13)

)->p

p

p_file = paste0(analysis_path,"/es_score.Th_cell_CD4.pdf")

ggsave(p_file,p,width = 12,height =5)

#Figrue8

library(monocle3)

t_rds_file <- "7.Trajectory/T_cell/tcell.rds"

t_rds <- readRDS(t_rds_file)

data.frame(id = rownames(t_rds@assays$integrated@data),

gene_short_name = rownames(t_rds@assays$integrated@data),

num_cells_expressed = rowSums(t_rds@assays$integrated@data != 0))->gene_annotation

cds <- new_cell_data_set(t_rds@assays$integrated@data,

cell_metadata = t_rds@meta.data,

gene_metadata = gene_annotation)

cds <- preprocess_cds(cds, num_dim = 50)

cds <- align_cds(cds, alignment_group = "projectID")

cds <- reduce_dimension(cds)

plot_cells(cds, label_groups_by_cluster=FALSE,

color_cells_by = "celltype",

#group_label_size = 3,

label_cell_groups = FALSE

)+

theme_classic()->p

p_file <- "6.Trajectory/T_cell/trajectory.pdf"

ggsave(p_file,p,width = 9)

cds <- learn_graph(cds)

plot_cells(cds, color_cells_by = "celltype",

label_groups_by_cluster = FALSE,

label_leaves = TRUE,

label_branch_points = TRUE,

graph_label_size = 1.5)

cds <- order_cells(cds,root_pr_nodes = c('Y_1','Y_1013','Y_1036','Y_121','Y_680'))

plot_cells(cds,

color_cells_by = "pseudotime",

label_cell_groups = FALSE,

label_leaves = FALSE,

label_branch_point = FALSE,

graph_label_size = 1.5)+theme_classic()->p

p_file <- "6.Trajectory/T_cell/pseudotime.pdf"

ggsave(p_file,p,width =7)

#specific gene

cds_pr_res <- graph_test(cds, neighbor_graph = "principal_graph",cores = 4)

#Figrue9

#TCR analysis

sample_list <-c("GSM4952970_P47_Tumor","GSM4952971_P55_Tumor","GSM4952972_P57_Tumor","GSM4952973_P57_Blood","GSM4952974_P58_Tumor",

"GSM4952975_P58_Blood","GSM4952976_P60_Tumor","GSM4952977_P60_Juxta","GSM4952978_P60_Blood","GSM4952979_P61_Tumor",

"GSM4952980_P61_Juxta","GSM4952981_P61_Blood")

sample_list_brief <-c("P47_Tumor","P55_Tumor","P57_Tumor","P57_Blood","P58_Tumor",

"P58_Blood","P60_Tumor","P60_Juxta","P60_Blood","P61_Tumor",

"P61_Juxta","P61_Blood")

tcr_df <- data.frame()

nb=6

for(i in 1:12){

tcr_file <- paste0("1.data/GSE162499/",

sample_list[i],"_filtered_contig_annotations.csv.gz")

tcr_df_tmp <- readr::read_csv(tcr_file)%>%

mutate(sample = sample_list_brief[i])%>%

mutate(barcode_corr = paste0(barcode,"_",nb))

rbind(tcr_df,tcr_df_tmp) -> tcr_df

nb = nb+1

}

data.frame(cele_id = Cells(rds),sample = rds$orig.ident)->rds_ids

separate(rds_ids,cele_id,into = c("barcode","rank"),sep = "_")->rds_ids

tcr_df%>%

inner_join(rds_ids)->tcr_df_1

subset(rds, cells = intersect(tcr_df_1$cell_id,Cells(rds)))->rds_tcr

DimPlot(rds_tcr,reduction = "umap", group.by = "celltype",label = TRUE, repel = TRUE,split.by="sample_class")->p

p_file <- "10.tcr/TCR.detect.umap.split.pdf"

ggsave(p_file,p,width = 12)

data.frame(barcode = Cells(rds_tcr),celltype = rds_tcr$celltype,sample_class = rds_tcr$sample_class)%>%

separate(barcode,into = c("barcode","barcode_fix"),sep = '_')%>%

left_join(tcr_df)->tcr_anno

ggplot(tcr_anno,aes(x = sample_class,y = cell_freq,fill = celltype))+

geom_bar(stat="identity",stack = "dodge")+

scale_fill_manual(values = clustcol[1:15])+

theme_classic()+

theme(

axis.text.x=element_text(color = "black", size=12),

axis.text.y=element_text(color = "black", size=7),

axis.title.x=element_text(color = "black", size = 13,face = "bold"),

axis.title.y=element_text(color = "black", size = 13,face = "bold")

)->p

p_file <- "10.tcr/population.bar.pdf"

ggsave(p_file,p)

ggplot(tcr_anno_brief,aes(x = celltype,fill = sample_class))+

geom_bar(position = position_dodge())+

scale_fill_manual(values = c("#FFFF00","#FF0033"))+

ylab("Contigs number")+

theme_classic() +

theme(

axis.text.x=element_text(color = "black", size=12,angle = 30,hjust = 1),

axis.text.y=element_text(color = "black", size=12),

axis.title.x=element_text(color = "black", size = 13,face = "bold"),

axis.title.y=element_text(color = "black", size = 13,face = "bold")

)->p

p_file <- "10.tcr/tcr.contig.num.pdf"

ggsave(p_file,p,width = 10,height =5)

ggplot(tcr_anno_brief,aes(x = celltype,y = length,fill = sample_class))+

geom_boxplot()+

scale_fill_manual(values = c("#FFFF00","#FF0033"))+

theme_classic() +

theme(

axis.text.x=element_text(color = "black", size=12,angle = 30,hjust = 1),

axis.text.y=element_text(color = "black", size=12),

axis.title.x=element_text(color = "black", size = 13,face = "bold"),

axis.title.y=element_text(color = "black", size = 13,face = "bold")

)->p

p_file <- "10.tcr/tcr.length.pdf"

ggsave(p_file,p,width = 10,height =5)
