# Supplementary material for: Metabolic and senescence characteristics associated with the immune microenvironment in non-small cell lung cancer: insights from single-cell RNA sequencing
Source: Aging (Albany NY). 2023 Oct 26;15(20):11571–87. doi: 10.18632/aging.205146 (PMC10637824; doi:10.18632/aging.205146)
Supplement: Supplementary Table 1 [file aging-15-205146-s002.pdf]

SUPPLEMENTARY TABLE

Supplementary Table 1. Cell annotation marker gene list.

Cell type	Marker genes
B cell	MS4A1, CD79A, CD79B, IGHD, CD19, CD74, HLA-DRA, CXCR4
Cancer cell	EPCAM+, CDH1+, CAPS-, SNTN-
Cytotoxic T cell	KLRC1, KLRD1, GZMB, PRF1
Endothelial cell	CLDN5, VWF, PECAM1
Epithelial cell	CAPS, SNTN, CLDN18, AQP4, FLOR1
Macrophage	C1QA, MRC1, MARCO, CD68, CD163, APOE
Macrophage: M2	MRC1, CD163, TGFB1, IL10, FN1
Monocyte	CD14, FCN1, FCGR3A, VCAN
Naive T cell (CD4-CD8-)	CCR7, LEF1, SELL, TCF7
T cell (CD4+CD8+)	CD2, CD3D, TRAC, TRBC2, CD4, CD8A, CD8B
Helper T cell (CD4+)	KLRB1, RORC
Regulatory T cell (CD4+)	FOXP3, IL2RA, CTLA4, IKZF2, CD4
